# Dihydromyricetin/Protein Pickering Emulsions: Interfacial Behavior, Rheology, and *In Vitro* Bioaccessibility

**DOI:** 10.3390/foods14142520

**Published:** 2025-07-18

**Authors:** Shengqi Mei, Lei Dou, Kaixuan Cheng, Guangqian Hou, Chi Zhang, Jianhui An, Yexing Tao, Lingli Deng, Longchen Shang

**Affiliations:** 1 College of Biological and Food Engineering, Hubei Minzu University, Enshi 445000, China; meishengqi0509@163.com (S.M.); doulei0119@163.com (L.D.); 17335897915@163.com (K.C.); 202330386@hbmzu.edu.cn (G.H.); zhtzu@163.com (C.Z.); saiwangan@163.com (J.A.); 2023060@hbmzu.edu.cn (Y.T.); 2019040@hbmzu.edu.cn (L.D.); 2 Hubei Key Laboratory of Selenium Resource Research and Biological Application, Hubei Minzu University, Enshi 445000, China

**Keywords:** dihydromyricetin complex, Pickering emulsion, delivery system, active substances

## Abstract

Protein-polyphenol-based delivery vehicles are effective strategies for encapsulating bioactive compounds, thereby enhancing their solubility and bioaccessibility. In this study, dihydromyricetin/soy protein isolate (DHM/SPI) complexes were used as emulsifiers to prepare Pickering emulsions for DHM delivery. The results show that DHM and SPI form negatively charged complexes through hydrogen bonding, and the complex size decreases and stabilizes with increasing DHM addition. The size of the emulsion droplets was inversely related to the concentration of DHM addition (*c*), particle concentration (*w*), and ionic strength (*i*). Conversely, the increasing oil phase concentration (*φ*) was positively correlated with droplet size. The CLSM results confirmed the expected oil-in-water emulsion, while the rheological behavior of the Pickering emulsion highlighted its elastic, gel-like network structure and non-Newtonian fluid properties. Moreover, DHM effectively slowed lipid oxidation in the emulsion, and the bioaccessibility of DHM reached 33.51 ± 0.31% after *in vitro* simulated digestion. In conclusion, this emulsion system shows promising potential for delivering DHM and harnessing its bioactive effects.

## 1. Introduction

Dihydromyricetin (DHM), a typical flavonoid compound, possesses potent antioxidant properties due to its unique pyran ring and polyhydroxy structure. Specifically, the hydroxyl groups on its B and C rings can effectively capture and scavenge various highly reactive free radicals, while also boosting the activity of antioxidant enzymes in the body, further enhancing antioxidant capacity [1]. Moreover, DHM also exhibits various pharmacological and biological activities, including liver protection, anti-inflammatory and antibacterial properties, as well as neuroprotection, making it a promising candidate for diverse applications in health management and disease prevention [2]. However, the limitations of DHM, including poor water solubility, susceptibility to degradation during gastrointestinal digestion, and low bioavailability, restrict its effectiveness and applications [3]. In recent years, Pickering emulsions have emerged as effective carriers for the delivery and encapsulation of functional agents and have gained widespread use in the food and pharmaceutical industries. Researchers commonly use biomolecules such as proteins [4], polysaccharides [5], and flavonoids [6] to stabilize Pickering emulsion systems. These particles form a stable film at the oil–water interface, preventing oil droplet aggregation and phase separation.

Soybean isolate protein (SPI) is a natural surfactant known for its low molecular flexibility and strong spatial rigidity. Near its isoelectric point, SPI reduces oil–water interfacial tension, promoting the dispersion of oil droplets in the continuous phase. This creates a stable interfacial membrane between protein particles and oil droplets, aiding emulsification and stabilizing classical emulsions [7]. SPI also stabilizes Pickering emulsions by forming a dense interfacial film at the oil–water interface through electrostatic adsorption and spatial resistance. This stabilizes the emulsion system and reduces the interfacial tension, effectively preventing droplet breakage, aggregation, and consolidation [8,9,10]. It has been reported that noncovalent interactions between SPI and polyphenol promote complex formation, which is helpful to stabilize aqueous–oil systems. Upon shear dispersion, these nanocomplexes absorb onto the oil–water interface, forming an interfacial film around the oil droplets and confirming the formation of Pickering emulsions [11]. In our study, this was observed using Confocal Laser Scanning Microscopy (CLSM), and the adsorption of the complexes at the water–oil interface proved that the preparation of DHM/SPI stabilized Pickering emulsion was successful. Emulsions stabilized using most protein-based Pickering particles, such as SPI, are usually oil-in-water emulsions. This characteristic has been confirmed by several studies on protein-based Pickering emulsions [12,13]. In addition, the gelling properties of proteins allow them to act as thickeners, forming a network structure within food systems that improves texture, viscosity, and mouthfeel. They also aid emulsion stabilization [14,15,16]. When proteins are used alone to stabilize Pickering emulsions, their sensitivity to pH, ionic strength, and temperature, as well as their tendency to aggregate or denature under varying conditions, makes it difficult to achieve sufficient emulsion stability. This limits their application in food emulsions [17,18,19]. However, the stability of proteins in emulsions can be enhanced by preparing complexes with other substances. To overcome this limitation, researchers are modifying proteins by incorporating organic substances, such as polysaccharides [20], polyphenols [11,21], and nucleotides [22], as well as inorganic substances [23]. These modifications, achieved through covalent or non-covalent interactions, help to maintain a stable and homogeneous system. The innovation potential of raw materials is immense, offering a wider range of options for developing Pickering emulsions and opening limitless applications.

Previous studies have highlighted the potential of flavonoids as Pickering particles to enhance the stability of O/W Pickering emulsions [24]. For instance, DHM can act as a standalone emulsifier to stabilize Pickering emulsions [25], and it can also be combined with high-amylose corn starch (HCS) composite particles [26] or cellulose nanofibers [27]. In these combinations, the composite particles enhance the stability of the emulsions while improving the overall functionality of the system. Studies have shown that non-covalent complexes of proteins and polyphenols can effectively stabilize the oil-in-water systems of Pickering emulsions [28]. In the presence of polyphenolic substances, there is a competitive adsorption of proteins and polyphenols at the interface. The interfacial interaction, driven by hydrogen bonding in protein/polyphenol complexes, enhances the stability of the protein in colloid system [29].

In this study, Pickering emulsions stabilized by non-covalent complexes of SPI and DHM were used for DHM delivery. The physicochemical properties and formation mechanisms of DHM/SPI complexes, formed with different DHM concentrations, were investigated. Pickering emulsions were prepared under various conditions, and their stability and structure were characterized at the mechanistic level. Additionally, the bioactivity of DHM was verified to delay lipid oxidation in Pickering emulsions. The impact of the Pickering emulsion on DHM’s retention rate and its bioaccessibility during the gastric and intestinal digestion was assessed. The results of this study offer new insights into the potential of protein-polyphenol complexes for stabilizing Pickering emulsions and delivering poorly soluble functional compounds.

## 2. Materials and Methods

### 2.1. Materials

Dihydromyricetin (DHM) was purchased from Shaanxi Xinpai Biotechnology Co., Ltd. (98%, Xi’an, China), soybean oil from Yihaijiali Jinlongyu Food Group Co., Ltd. (Shanghai, China), soy protein isolate (SPI, >90%) from Shanghai Yuanye Biotechnology Co., Ltd. (Shanghai, China), Nile Red, Nile Blue A, 1,1-diphenyl-2-picrylhydrazyl (DPPH), and 2,2-azinobis (3-ethyl-benzothiazole-6-sulfonic acid) diammonium salt (ABTS), and other reagents were purchased from Shanghai Yuanye BioTechnology Co., Ltd. (Shanghai, China).

### 2.2. Preparation of DHM/SPI Complexes

5 g of SPI powder was dissolved in 200 mL of purified water and hydrated overnight at 4 °C. Then, varying concentrations of DHM alcohol solution were added to the system under magnetic stirring, with DHM masses of 0 g, 0.25 g, 0.5 g, 0.75 g, and 1.0 g (corresponding to 0%, 5%, 10%, 15%, and 20% of the mass of SPI, respectively). The reaction was carried out for 2 h to obtain SPI solution mixtures containing 0%, 5%, 10%, 15%, and 20% DHM. The resulting mixtures were freeze-dried to yield DHM/SPI complexes, labeled as 0% DHM/SPI, 5% DHM/SPI, 10% DHM/SPI, 15% DHM/SPI, and 20% DHM/SPI.

### 2.3. Characterization of DHM/SPI Complexes

#### 2.3.1. Chromaticity

The color parameters of the DHM/SPI complex powder were measured at six random points using a colorimeter (CS-820N, Hangzhou CHNSpec Technology Co., Ltd., Hangzhou, China), recording L* (luminance), a* (red to green), and b* (yellow to blue). The total color difference (∆*E*) and whiteness index (*WI*) of the complex were calculated using the following equations:

$$\Delta E=[{{{(\Delta L}^{*})}^{2}+{{(\Delta a}^{*})}^{2}+{{(\Delta b}^{*})}^{2}]}^{\frac{1}{2}}$$

$$WI=100-[{{{(100-L}^{*})}^{2}+{{(a}^{*})}^{2}+{{(b}^{*})}^{2}]}^{\frac{1}{2}}$$

where ΔL*, Δa*, and Δb* represent the differences between the standard color plate and the complex.

#### 2.3.2. Contact Angle

Following the method reported by Zhang et al. [30], the DHM/SPI complex powder was pressed into a thin sheet, and a drop of deionized water was added perpendicularly to its surface. Once equilibrium was reached, the shape of the droplet was recorded using a contact angle meter (Dataphysics OCA 20, Filderstadt, Germany) with a camera. The contact angle (θ) was determined using the Laplace–Young equation.

#### 2.3.3. Particle Size and Zeta Potential

The DHM/SPI complex was diluted to 0.1 mg/mL, and the particle size and potential values were determined using a particle size potentiostat (Zetasizer Nano-ZS Zen 3600, Malvern, UK) at 25 °C.

#### 2.3.4. Fourier Transform Infrared Spectroscopy (FTIR)

The samples were placed on a Fourier infrared (IR) spectrometer with a total reflection attachment for FTIR spectral scans. The FTIR spectra of the DHM/SPI complex and DHM were collected, deconvoluted by Omnic software, and analyzed using PeakFit v4.12 software for smoothing and second-order derivatives. The spectral range was 4000–400 cm^−1^ with a resolution of 4 cm^−1^ and 32 s.

#### 2.3.5. X-Ray Diffraction (XRD)

X-ray diffraction (XRD) spectra of the DHM/SPI complex and DHM were obtained using an X-ray diffractometer (Shimadzu XRD-7000, Kyoto, Japan). The scanning parameters were set to 30 mA and 40 kV, the scanning range was 5–60°, the step width was 0.02°, and the scanning rate was 5° min^−1^.

#### 2.3.6. Thermogravimetric Analysis (TG)

The thermal stability of the DHM/SPI complex, SPI, and DHM was evaluated using a thermogravimetric analyzer (SII TG/DTA 6300, Tokyo, Japan) with nitrogen as the purge gas (100 mL min^−1^). The samples were placed in alumina dishes, and the temperature was raised from 35 to 600 °C at a rate of 10 °C/min.

#### 2.3.7. Molecular Docking Modeling of DHM and SPI

The 7S and 11S globin structures of SPI were downloaded from the Protein Data Bank (RCSB PDB) database. The structure of DHM was generated using Chem3D 20.0 software. Molecular docking between the 7S and 11S globin structures of SPI and DHM was performed using AutoDock Vina 1.2.0 program (Scripps Research, La Jolla, CA, USA). The docking results were evaluated based on the lowest binding energy and visualized using PyMOL software (DeLano Scientific LLC, South San Francisco, CA, USA).

### 2.4. Preparation of Pickering Emulsion Stabilized by DHM/SPI Complexes

Pickering emulsions were prepared using DHM/SPI composite particles as emulsifiers, based on the method of Geng et al., with modifications [26]. Specific amounts of DHM/SPI composite particles (c, 0–20% DHM, *w/w*) were dispersed in aqueous NaCl solutions (*i*) at concentrations ranging from 0 to 400 mM. The aqueous dispersions were mixed with soybean oil at different oil phase volume fractions (*φ*, 40–80% *v*/*v*). The concentration of composite particles (*w*) in the mixture was between 0.5% and 4% (*w*/*v*). The mixture was subjected to high-speed shearing using a digital high-speed homogenizer (IKA Ultra-Turrax T25, Freiburg, Germany) at 12,000 rpm for 3 min. The produced emulsions were grouped separately (Table 1) and transferred to glass bottles.

### 2.5. Characterization and Bioactivity Determination of Pickering Emulsions Stabilized by DHM/SPI Complexes

#### 2.5.1. Zeta Potential

Determination of zeta potential using freshly prepared emulsions. A total of 400 µL of the Pickering emulsion was diluted with ultrapure water to a final volume of 4 mL. The zeta potential was measured using a particle size potentiostat (Zetasizer Nano-ZS Zen 3600, Malvern, UK).

#### 2.5.2. Optical Microscopy

The microstructure of the newly formed emulsion was examined using a polarized light microscope. Images were captured with a digital camera, and the emulsion size was analyzed using Image J 1.54f software (National Institutes of Health, Bethesda, MD, USA).

#### 2.5.3. Confocal Laser Scanning Microscopy (CLSM)

The microstructure of the emulsions was examined using a laser confocal scanning microscope (Olympus FV3000, Olympus Corporation, Tokyo, Japan). The emulsions were stained with 0.1% Nile red and 0.1% Nile blue at room temperature, protected from light. After staining, the emulsions were transferred to slides, covered with a coverslip, and analyzed under the microscope. The dyes were excited at 535 nm (Nile red) and 630 nm (Nile blue) to visualize the emulsion microstructure.

#### 2.5.4. Creaming Index (CI)

Fresh emulsions were stored in sealed glass bottles at 25 °C for 15 d. The change in height of the emulsions due to delamination was recorded. The CI was calculated as follows:

CI (%)=HSHT×100

where *H_S_* is the height of the lower clear liquid layer, and *H_T_* is the total height of the emulsion.

#### 2.5.5. Rheological Behavior

The rheological properties of the emulsion were measured using a rotational rheometer (TA Instruments LSD AR2000ex, New Castle, DE, USA) with 40 mm parallel steel plates and a measurement gap of 1.00 mm. Following previously reported methods, strain and frequency scans and steady-state shear were performed on the emulsions to determine their rheological behavior [31,32]. Additionally, we adjusted the fixed strains to the linear viscoelastic region (LVR) as measured in this study. The linear viscoelastic region was determined by amplitude sweeping the strain from 0.1 to 100% at a fixed frequency of 1 Hz. Frequency sweeps with angular frequencies ω of 1–100 rad/s were performed at a constant strain of 1%. Finally, the apparent viscosity was determined at shear rates from 1 s^−1^ to 100 s^−1^.

#### 2.5.6. Lipid Peroxidation

The prepared emulsions were subjected to accelerated oxidation at 40 °C to evaluate the effect of DHM addition on their oxidative stability by determining the amounts of primary and secondary oxidation products.

Primary oxidation products: According to the method of Wang et al. [33], 0.3 mL of emulsion was mixed with 1.5 mL of isooctane/isopropanol (3:1, *v*/*v*), then centrifuged at 10,000 rpm for 5 min. A total of 200 μL of the supernatant was collected, to which 2.8 mL of methanol/n-butanol (2:1, *v*/*v*), 15 μL of ferrous iron solution (composed of 0.132 M BaCl_2_ and 0.144 M FeSO_4_), and 15 μL of 3.94 M ammonium thiocyanate solution were added. After a 20 min dark reaction, the absorbance was measured at 510 nm, and the standard curve was established using hydrogen peroxide.

Secondary oxidation products: A mixture of 15% (*w*/*v*) trichloroacetic acid and 0.375% (*w*/*v*) thiobarbituric acid in 0.025 M HCl was prepared, following the method of Yi et al. [34] 1 mL of emulsion was mixed with 2 mL of thiobarbituric acid (TBA) solution, heated in a boiling water bath for 20 min, then cooled and filtered after centrifugation at 4000 rpm for 10 min. The absorbance was measured at 532 nm, and the standard curve was established using 1,1,3,3-tetraethoxypropane.

#### 2.5.7. *In Vitro* Simulated Digestion

Simulated Gastric Digestion: According to the method of Gao et al. [35], a simulated gastric digestion solution was prepared by dissolving 3.2 mg/mL pepsin, 2 mg/mL NaCl, and 7 mL/L HCl. This solution was mixed in equal proportions with a sample of Pickering emulsion stabilized by various DHM/SPI complexes and pure SPI, and the pH was adjusted to 2.0. The mixture was placed on a constant temperature shaking table at 37 °C and shaken at 100 rpm for 2 h. The absorbance of DHM in the gastric digested solution was then measured at 292 nm.

Simulated Intestinal Digestion: A simulated enteric digestion solution was prepared using 36.7 mg/mL CaCl_2_ and 218.7 mg/mL NaCl. A separate 54 mg/mL bile salt solution was prepared, and 24 mg/mL trypsin and 24 mg/mL lipase were dissolved in phosphate buffer (pH 7.0) and set aside. After completing the gastric digestion, the pH of the mixture was adjusted to 7.0. Then, 2.5 mL of trypsin, 2.5 mL of lipase, 3.5 mL of bile salts, and 1.5 mL of simulated intestinal fluid were added. The mixture was placed on a constant-temperature shaker at 37 °C and shaken at 100 rpm for 2 h. The absorbance of DHM in the intestinal digest was measured at 292 nm.

The retention of DHM after gastric and intestinal digestion and the bioaccessibility of DHM were determined and calculated according to the method of Huang et al. [36]:

$$\mathrm{DHM}\;\mathrm{gastric}\;\mathrm{digestion}\;\mathrm{retention}\;(\%)=\frac{C_{T1}}{C_{0}}\times 100$$

$$\mathrm{DHM}\;\mathrm{intestinal}\;\mathrm{digestion}\;\mathrm{retention}\;(\%)=\frac{C_{T2}}{C_{T1}}\;\times 100$$

$$\mathrm{DHM}\;\mathrm{bioaccessibility}\;(\%)=\frac{C_{micelle}}{C_{0}}\;\times 100$$

where *C_0_* is the DHM content in the initial digestive system; *C_T_*_1_ is the DHM content in the mixture after gastric digestion, and *C_T2_* is the DHM content in the mixture after intestinal digestion; where *C*_micelle_ is the contents of DHM solubilized in the micelles.

### 2.6. Statistical Analysis

Each experiment in this study was conducted in triplicate, and subsequent statistical analysis and graphical representation were conducted using the software packages SPSS 25.0 and Origin 2021. The results were presented as the mean  ±  standard deviation (SD). Analysis of variance (ANOVA) was conducted, followed by comparisons of means using Duncan’s multiple range test. Significance was determined at *p*  <  0.05.

## 3. Results and Discussion

### 3.1. Chromaticity Analysis of DHM/SPI Complexes

**Figure 1:**
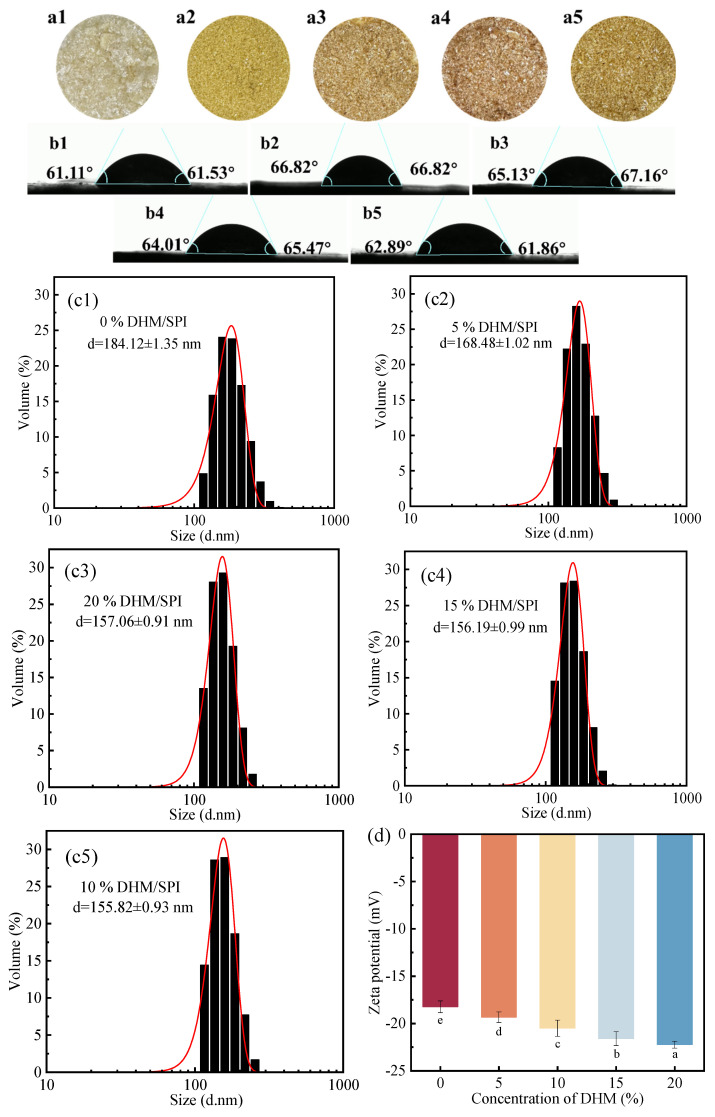
(**a1**–**a5**) Effect of different DHM additions on the color of complexes; (**b1**–**b5**) contact angles of DHM/SPI complexes with different DHM additions; (**c1**–**c5**) particle size of complexes with different DHM additions; (**d**) zeta potential of complexes with different DHM additions.

DHM, containing conjugated double bonds and phenolic hydroxyl groups, can absorb specific wavelengths of light and impart distinct colors to complexes under certain conditions. The stability of these colors is closely linked to the antioxidant activity of the complexes. The addition of DHM affects the color of the composites (Figure 1a1–a5), significantly lowering the L* value of the samples compared to the control group (Table 2). This change is primarily due to the polyhydroxyl structure of DHM, where hydroxyl groups are oxidized into o-quinones with electrophilic properties under aerobic conditions. These o-quinones can undergo polymerization to form a brown complex with a high molecular weight. Furthermore, the o-quinones can react with nucleophilic groups (such as thiols and amino groups) in SPI, leading to the formation of various pigments that deepen the color and decrease the brightness of the sample [37].

The ∆*E* index is commonly used to represent the color difference between the control and sample groups. The ∆*E* value for the complex with the highest DHM addition was 57.47 ± 0.42, which was significantly different from that of the 0% DHM/SPI complex (pure SPI), but not significantly different from the 15% DHM/SPI complexes. This suggests that the ∆*E* value gradually decreased with increasing DHM addition, with the difference becoming less significant among these DHM/SPI complexes. This might be attributed to the fact that DHM is dissolved in ethanol, which has reducing properties. Ethanol can reduce the o-quinones to a colorless o-diphenol precursor, thus inhibiting the formation of o-quinones and reversing the color change [38]. At high DHM concentrations, more o-quinones are produced, promoting the color reversal reaction and decreasing pigment production.

### 3.2. Contact Angle Analysis of DHM/SPI Complexes

The contact angle analysis of DHM/SPI composite particles provides valuable insight into the emulsion type that the particles can stabilize. The contact angle is directly related to the hydrophilicity or lipophilicity of the particles, which in turn influences the type of emulsion formed. Typically, particles with a contact angle between 15° and 90° are more hydrophilic, stabilizing oil-in-water (O/W) emulsions, while particles with a contact angle between 90° and 180° are more lipophilic, stabilizing water-in-oil (W/O) emulsions [39].

The water contact angle is a key indicator of the hydrophobicity of a solid surface. In this study, the hydrophobic benzene ring of the DHM molecule modified the SPI through the hydrophobic effect, enhancing the hydrophobicity of the complex and increasing the contact angle. Additionally, DHM induced partial unfolding of the SPI structure, exposing more hydrophobic amino acids, which facilitated further interactions. This altered the chemical composition and arrangement of the protein surface, orienting the hydrophobic regions outward. Consequently, the surface hydrophobicity of the complex was further enhanced, as evidenced by a significant increase in the contact angle (θ) (Figure 1b1–b5) [40]. As the concentration of DHM increased, the contact angle of DHM/SPI particles initially increased and then decreased. This behavior was attributed to the increasing concentration of DHM, where hydrophilic groups, such as hydroxyl groups, covered the protein surface, thereby enhancing the polarity and hydrophilicity of the surface. Simultaneously, the exposure of hydrophobic groups in the protein increased, providing more sites for both inter- and intramolecular hydrophobic interactions. However, when the DHM concentration reached a certain threshold, non-covalent bonding, primarily hydrogen bonding, became the dominant interaction. As a result, the primary force maintaining the system shifted from hydrophobic interactions to hydrogen bonding, leading to a decrease in hydrophobicity and an increase in hydrophilicity, which caused a reduction in the contact angle (θ) and a decrease in surface hydrophobicity. In the study of konjac glucan (KGM) and myofibrillar protein (MP) complexes, an interesting phenomenon similar to the contact angle behavior of the DHM/SPI complexes described above emerged [41]. Although the contact angles of the DHM/SPI complexes changed with an increase in DHM content, this change was minimal, indicating that the particles’ hydrophobicity did not change significantly and their hydrophilicity remained strong. This was conducive to stabilizing oil-in-water (O/W) emulsions. The contact angle is highest at a DHM concentration of 5%, indicating that it is more effective at stabilizing oil-in-water Pickering emulsions. DHM has poor solubility on its own, but the DHM/SPI complex exhibits enhanced hydrophilicity. This allows it to be wet by water, forming a hydrated layer that improves the solubility, dispersion, and overall utilization of DHM.

### 3.3. Particle Size and Zeta Potential Analysis of DHM/SPI Complexes

By determining the particle size and zeta potential of DHM/SPI complexes, we investigated the effects of varying DHM concentrations on the size and surface charge of the complexes (Figure 1c1–c5,d). The results showed that the particle size of the complexes gradually decreased and stabilized as the DHM concentration increased. The addition of DHM altered the conformation of the protein molecules, reducing spatial potential resistance and consequently decreasing the particle size. Moreover, we observed that the particle size of the DHM/SPI complexes was smaller than that of soybean isolate protein alone. Generally, a smaller particle size correlates with greater stability of the complex in the system. Non-covalent interactions between DHM and SPI result in the encapsulation of DHM within the SPI matrix, as supported by molecular docking analysis. This encapsulation exposes the intrinsic negative charge of SPI, contributing to the overall negative zeta potential of the complex. As the concentration of DHM increases, these interactions alter the charge distribution on the protein surface. Specifically, the introduction of DHM contributes additional negatively charged hydroxyl groups, thereby enhancing the net negative charge of the protein. This progressive increase in surface negativity with higher DHM concentrations indicates improved electrostatic stabilization of the complexes. A similar phenomenon was reported by Tian et al. in their study on the complex of pea isolate protein and naringenin, where increasing naringenin concentration gradually enhanced and stabilized the absolute values of both the particle size and zeta potential of the complexes [42].

### 3.4. XRD Analysis

**Figure 2:**
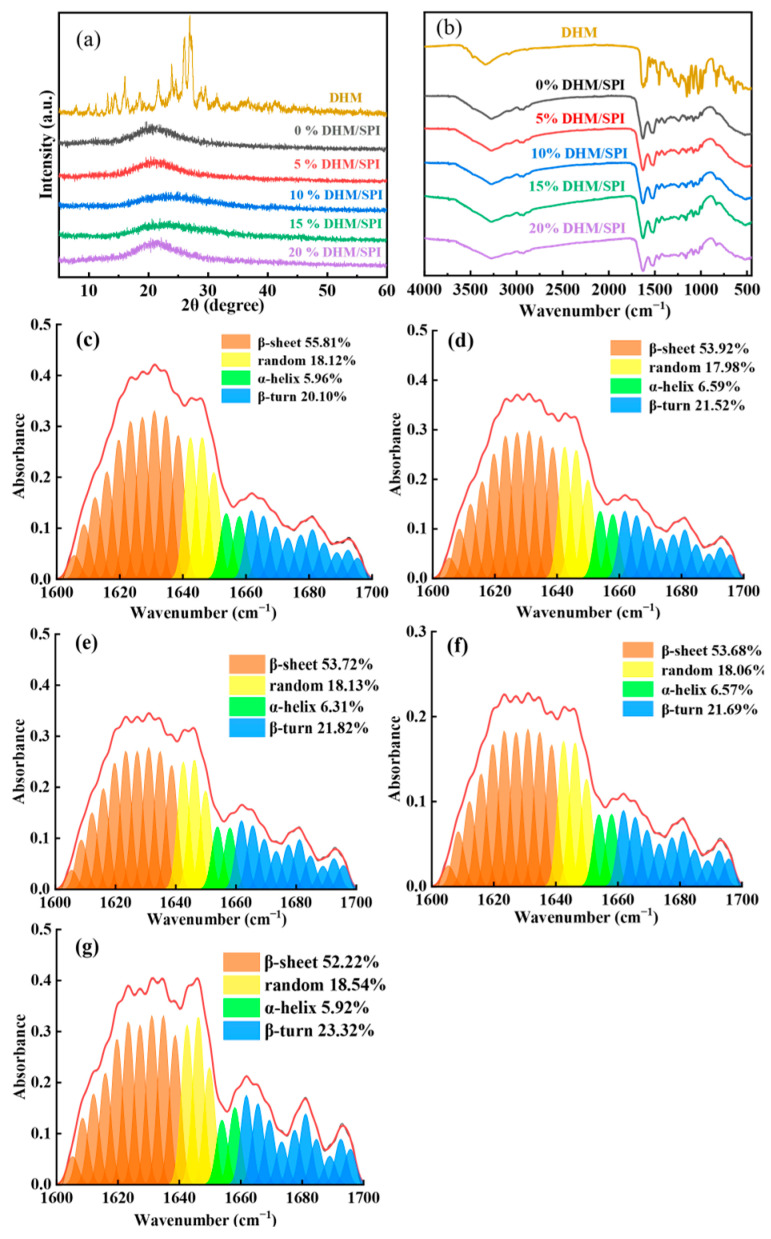
(**a**) X-ray diffractograms of DHM/SPI complexes with different DHM mass fractions versus DHM. (**b**) FTIR spectra of DHM/SPI complexes with different DHM mass fractions versus DHM; (**c**–**g**) deconvolution and curve fitting bands of FTIR spectra of amide I bands of DHM/SPI complexes, with DHM additions from 0% to 20%.

The X-ray diffraction (XRD) analysis of DHM and SPI complexes reveals valuable insights into their structural interactions (Figure 2a). DHM exhibits distinct sharp peaks at specific 2θ values around 15° and between 20 and 30°, indicating its crystalline nature typical of flavonoid compounds [43]. These sharp peaks signify well-ordered crystalline structures, characteristic of DHM’s solid-state arrangement. SPI, on the other hand, displays a V-shaped structure with a significant diffraction peak at 22.83°, representing an amorphous structure typical for natural proteins. The V-shape in its diffraction pattern suggests a certain degree of disorder in the protein’s arrangement [44]. When DHM interacts with SPI to form the DHM/SPI complex, the resulting diffraction pattern no longer shows the sharp peaks typical of DHM’s crystalline form. In contrast, the DHM/SPI complex preserved the amorphous structure of soybean protein isolate (SPI), with a broad and flat peak ranging from 15° to 30°. This indicated a strong noncovalent interaction between DHM and SPI, disrupting the original crystalline structure of DHM and preventing the formation of distinct diffraction peaks in these DHM/SPI complexes [45]. As a result, the diffraction peaks of DHM disappeared, confirming successful encapsulation within the SPI matrix. This encapsulation not only alters the physical state of DHM but also enhances its chemical properties, solubility, and stability. A similar phenomenon is observed in other complex systems. For instance, Ye et al. modified zein using hyaluronic acid (HA) and naringenin (Nar), forming Nar/Zein-HA composite nanoparticles [46]. The XRD pattern of these nanoparticles also showed an amorphous conformation, like the DHM/SPI complex, indicating that naringenin (Nar) is in an amorphous form within the composite. In general, amorphous structures are known to have superior water solubility and stability compared to their crystalline counterparts. This enhanced solubility and stability could improve the bioavailability of bioactive compounds, like DHM and naringenin, making them more effective in biological applications [47]. Nevertheless, during the preparation of the complexes, the uneven distribution of DHM in the SPI matrix and the high concentration of DHM in some regions due to uneven mixing or local concentration differences still revealed some weak DHM crystal peaks in the X-ray diffraction patterns of the DHM/SPI complexes.

### 3.5. FTIR and Secondary Structure Analysis

Fourier Transform Infrared (FTIR) spectroscopy provides insight into the molecular interactions and secondary structural changes in the DHM/SPI composite particles (Figure 2b). After adding DHM to SPI, a broad absorption peak at 3300 cm^−1^ appears in the FTIR spectrum. This peak corresponds to the N-H stretching vibration of the protein amide A band, suggesting that DHM is inducing hydrogen bonding with the protein [48]. This interaction indicates that DHM is likely engaging with the protein, contributing to changes in the secondary structure of SPI. As more DHM is added, there is a slight decrease in the wavenumber of this absorption peak, which is associated with changes in the hydrogen-bonding environment in the complex. The absorption peak arising from the stretching vibration of the protein carbonyl group (C=O) typically appears between 1600 and 1700 cm^−1^ and is a key feature of the amide I band in proteins. This region, in combination with the weak vibrations of the N-H and C-N bonds, provides information about the protein’s secondary structure. The Amide I band can be further subdivided into different regions, each corresponding to different types of secondary structures. Among them, the strong absorption of the amide I band at 1600–1640 cm^−1^ corresponds to β-sheet, at 1640–1650 cm^−1^ corresponds to random, at 1650–1660 cm^−1^ corresponds to α-helix, at 1660–1670 cm^−1^ corresponds to strong absorption as β-turn, and these four forms make up the secondary structure of the protein.

By applying back-convolution or second derivative processing to the amide I bands of DHM/SPI complexes with varying DHM concentrations, structural details of β-sheet, random, α-helix, and β-turn can be extracted for quantitative analysis of the proportions of different secondary structures in proteins (Figure 2c–g). The proportion of β-sheet decreases from 55.81% to 52.22% as DHM is added. The amount of β-turn increases from 20.10% to 23.32% with higher levels of DHM. These changes indicate a change in the secondary structure of the protein, with an increase in β-turn structures and a corresponding decrease in β-sheet, suggesting that DHM promotes the formation of more β-turns. The shift in the secondary structure is influenced by the polarity and charge distribution of the amino acid side chains in SPI. The introduction of DHM likely promotes the formation of β-turn structures, which are stabilized by hydrogen bonds and interactions with DHM. The β-turns are often found in proteins with polarity or charged residues that favor such conformations. The FTIR analysis reveals that the addition of DHM to SPI results in the formation of a hydrogen-bonded composite, leading to changes in the secondary structure of the protein [49]. Specifically, DHM induces a decrease in β-sheet and an increase in β-turn, suggesting that DHM interacts with SPI in a way that alters the protein’s conformation.

### 3.6. Thermogravimetric Analysis

**Figure 3:**
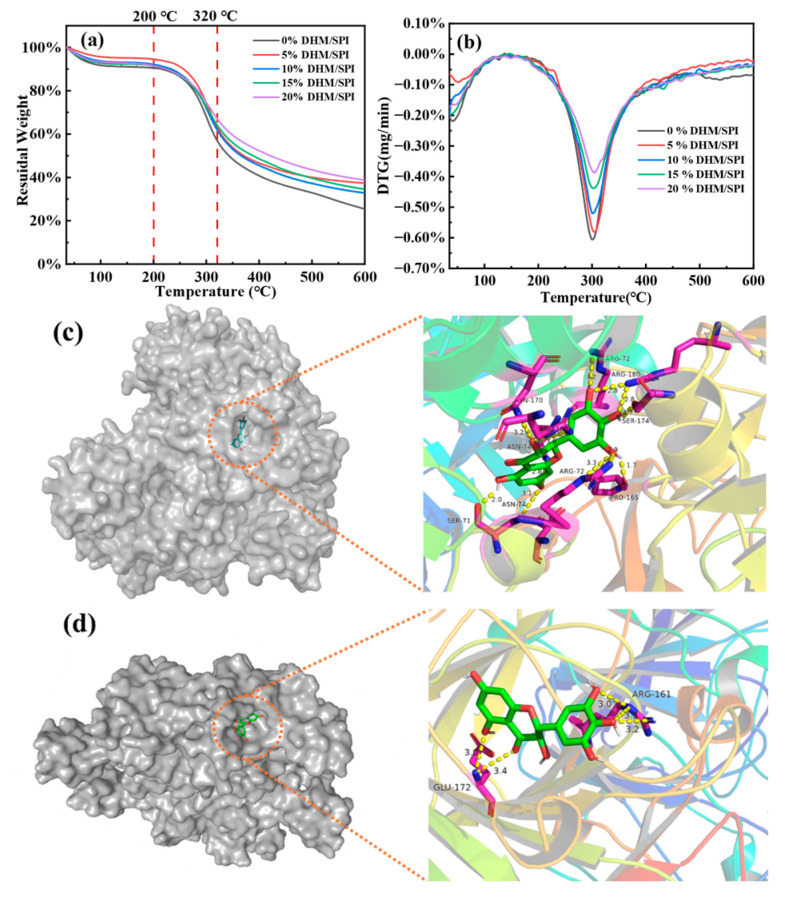
(**a**) Thermogravimetric analysis (TG) images of different DHM/SPI complexes with SPI. (**b**) Differential thermogravimetric analysis (DTG) images of different DHM/SPI complexes with SPI. (**c**) Molecular docking model of SPI’s 7s globulin and DHM; (**d**) molecular docking model of SPI’s 11s globulin and DHM.

We analyze the TG and DTG curves of the complexes to investigate the thermal stability of the DHM/SPI composite particles. The thermal stability of pure DHM, a natural polyphenolic compound, has previously been reported in the literature, with melting and decomposition occurring at 250 °C [50]. The mass loss of the DHM/SPI composite particles occurs in three distinct stages during heating (Figure 3a). The first weight loss stage was 35–200 °C, which is mainly attributed to the loss of free and bound water within the DHM/SPI complexes. The evaporation of water led to a slight loss in sample weight. This is a typical feature in composite materials containing water, as the heat drives off residual moisture. When the temperature reaches 200–320 °C, the thermal weight loss of the sample comes to the second stage, and the hydrogen bonds between DHM and SPI break, leading to the decomposition of the carbon molecular skeleton of the composite. The main structural components of the complex begin to degrade. The maximum decomposition rate occurs at 300 °C, which aligns with the peak temperature observed in the DTG curve (Figure 3b). As the concentration of DHM increases, the decomposition mass of the composite at the temperature of maximum decomposition rate decreases. This suggests that DHM affects the stability of the composite material, likely by influencing the degradation behavior of the protein structure. After increasing the temperature to 320–600 °C, the weight loss corresponds to the incomplete decomposition of the complex and the potential presence of impurities. This stage shows a slower and less significant weight loss compared to the previous two stages, indicating the remaining material is less prone to decomposition at higher temperatures. DHM/SPI complexes with different DHM mass fractions showed better thermal stability than pure SPI. The analysis suggests that the hydrogen bonding interactions between DHM and SPI play a crucial role in enhancing the thermal stability of the protein complex [51]. By forming these bonds, DHM likely helps to stabilize the protein structure, making it more resistant to thermal degradation. Similar findings have been reported in systems where tea polyphenols (TPs) were added to SPI. In those systems, the addition of TPs was shown to increase the degradation temperature, improving the thermal stability of vegetable protein composites [52].

### 3.7. Molecular Docking Modeling Analysis of DHM and SPI

To further investigate the binding site and type of interaction between the protein and the flavonoid, molecular docking was performed with the 7S and 11S globulins of SPI and DHM. The optimal docking conformation of the complex is shown in Figure 3c,d, where the globulin is represented by a curved strip-like structure, and the small-molecule flavonoid DHM is represented by a stick-like structure connected to it. The data indicate that the docking of DHM with SPI components, specifically 7S and 11S globulins, results in stable complexes. The lowest binding energies observed were −9.2 kcal/mol for the 7s globulin and −8.2 kcal/mol for the 11s globulin. These values suggest that DHM forms a more stable complex with the 7S globulin, which implies a higher stability or stronger interaction between DHM and 7S globulin.

The hydroxyl group of DHM binds to several amino acid residues on both globulins, forming hydrogen bonds. For 7S globulin, DHM interacts with Arg-72 (2.9 Å, 3.2 Å, 3.3 Å), Arg-180 (2.8 Å, 3.6 Å), Ser-71 (2.8 Å, 3.6 Å), and Ser-71 (2.8 Å, 2.6 Å). For 11S globulin, hydrogen bonds are formed with Ser-71 (2.0 Å), Ser-174 (1.7 Å, 2.8 Å), Asn-170 (3.2 Å), Pro-165 (1.7 Å), and Asn-74 (3.1 Å), in addition to Arg-161 (3.0 Å, 3.2 Å, 3.3 Å) and Glu-172 (3.0 Å, 3.4 Å). These interactions show that DHM primarily binds to SPI via hydrogen bonding, with a particularly strong interaction with multiple amino acid residues on the 7S globulin, notably Arg and Ser.

The nonpolar groups of polyphenols can spontaneously form multipoint hydrogen bonds with the amino acid residues of protein molecules, particularly in regions where the protein’s hydrophobic groups are concentrated. For example, the complex of Spirulina protein isolate with tannic acid involves hydrogen bonding, hydrophobic interactions, and other non-covalent forces such as π-stacking and salt bridges. Overall, hydrogen bonding remains the primary force in maintaining the stable interaction of the complex [21].

### 3.8. Formation of Pickering Emulsions of DHM/SPI Complexes and CI During Storage

**Figure 4:**
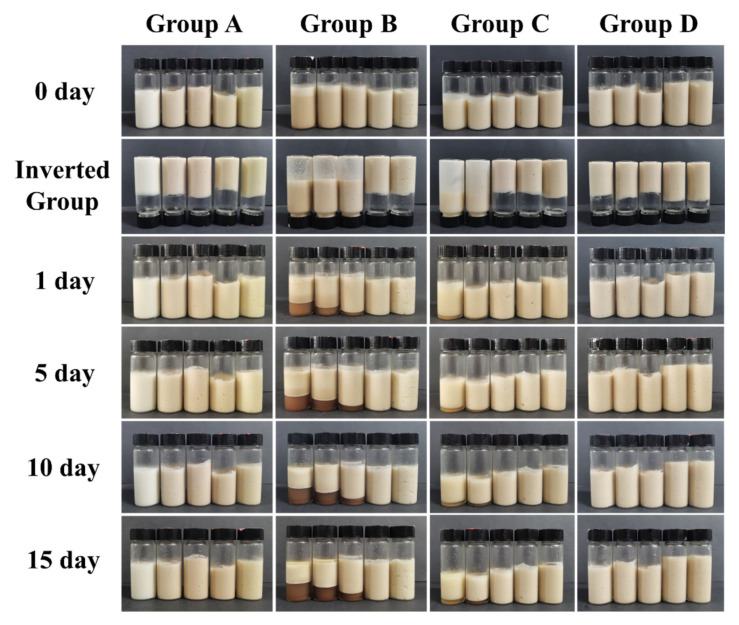
Appearance of Pickering emulsions prepared under different conditions during the storage period.

The formation of Pickering emulsion is influenced by various factors, including the DHM content (*c*), oil phase mass fraction (*φ*), composite particle addition (*w*), and ionic strength (*i*) of the composite particles. DHM/SPI composite particles are adsorbed at the water–oil interface, which allows the composite to act as Pickering particles. This is fundamental to the stabilization of Pickering emulsion systems. The emulsion stratification phenomenon occurs due to density differences and gravity. The larger oil droplets move upwards and separate from the emulsified phase. With an evolved stage of creaming, an oil layer forms on top of the emulsion [53]. To assess the stability of Pickering emulsions stabilized by the DHM/SPI complexes, samples were stored at 25 °C for 15 d and used to measure their creaming index (CI) (Figure 4).

The emulsion system can be stabilized when the complexes form a viscous emulsion at *w* = 2%, *i* = 0, and *φ* = 70% (Figure 4 Group A). Both the water–oil ratio and particle concentration affect the type and extent of intermolecular interactions in Pickering emulsion. When *φ* < 70%, emulsions formed at *c* = 5%, *w* = 2%, and *i* = 0 have a low viscosity and cannot withstand gravity when stacked simply (Figure 4 Group B). Initially, emulsions experienced rapid delamination during the pre-storage period; this rate of increase slowed over time and stabilized, with no significant change in CI after 10 d, indicating that molecular motion in the emulsion system had reached equilibrium. However, increasing the oil phase fraction to *φ* ≥ 70% enables the DHM/SPI stabilized emulsion system to form Pickering emulsions with higher viscosity. Continuing to increase the oil phase fraction achieves a high internal phase emulsion. This outcome is attributed to the higher oil content in the emulsion system, which causes the aggregation of small droplets. The intermolecular forces between these droplets increase the resistance to deformation, thereby enhancing the stability of the emulsion.

The complexes formed stable, viscous emulsions at *w* = 2%, *i* = 0, and *φ* = 70% (Figure 4, Group A). The water–oil ratio and particle concentration influence the type and extent of intermolecular interactions in Pickering emulsions. When *φ* < 70%, the gel-like structure of the emulsion could not form under the conditions of *c* = 5%, *w* = 2%, and *i* = 0 (Figure 4 Group B). The emulsions showed faster delamination during the pre-storage period. As storage time extended to the 10th day, the growth of the CI slowed and stabilized, indicating that the molecular motion within the emulsion system had reached equilibrium. The Pickering emulsion with a 40% oil phase fraction exhibited the highest CI values of 39.43 ± 0.42% and 48.55 ± 0.46% after 15 d, suggesting it was the least stable compared to the other emulsions (Table 3). When the oil phase fraction was increased to *φ* ≥ 70%, the DHM/SPI-stabilized emulsion system formed Pickering emulsions with higher viscosities. Further increasing the oil phase fraction led to the formation of stable high internal phase emulsions, which inhibited phase separation. This stability is attributed to the higher oil content, which causes the aggregation of tiny droplets and the intermolecular forces between the droplets, enhancing the deformation resistance of the emulsion [31].

Normally, increasing the concentration of Pickering particles aids in the formation and stabilization of the Pickering emulsion gel network. In this study, at lower concentrations of Pickering particles (*w* < 2 wt%), the system failed to form a gel-like structure, and phase separation occurred during storage. However, when the DHM/SPI content exceeded 2 wt%, phase separation was eliminated (Figure 4, Group C). The enhanced electrostatic repulsion between droplets, due to the increased spatial site resistance provided by DHM, contributed to the formation of a stable emulsion system. Ionic strength also influences emulsion stability through the repulsion of electrostatic interactions between particles. However, no significant difference was observed in the gelation effect of Pickering emulsions formed under different ionic strength conditions, and no noticeable phase separation occurred with increasing ionic strength (Figure 4, Group D). This suggests that the size of the formed droplets remained relatively stable and did not change significantly [54]. Moderate increases in ionic strength slightly improved the stability and viscosity of the Pickering emulsion. The stable emulsion of the SPI system with added DHM was only minimally affected by ionic strength, indicating that DHM addition could enhance the stability of SPI proteins under varying ionic conditions. This finding holds promise for food emulsion systems with high salt content, offering more potential for development in these applications.

As the storage time extended to 15 d, oil separation was observed in the upper layer of the emulsion, particularly in samples with a lower oil phase ratio and particle concentration. This phenomenon resulted from the aggregation and flocculation of oil droplets within the emulsion, leading to an increase in droplet size and a significant difference in density. This density disparity accelerated the upward movement of the precipitated oil. However, overall, the Pickering emulsions stabilized by the DHM/SPI complexes maintained stability for a longer period under the appropriate system conditions.

### 3.9. Optical Microscopy of Pickering Emulsion

**Figure 5:**
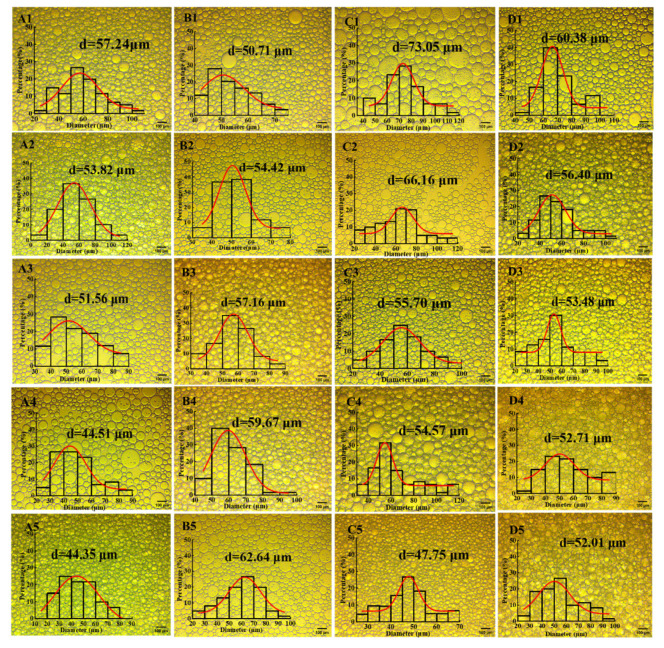
Optical micrographs and particle size distribution of emulsions prepared under different conditions; scale bar: 100 μm.

The droplet size of protein-based Pickering emulsions is typically influenced by environmental factors, such as the water–oil ratio, concentration, and ionic strength. Microscopic observation of the emulsions’ microstructure provides insight into how these factors affect droplet size (Figure 5, A1–D5). For Pickering emulsions stabilized by the DHM/SPI complex, the droplet size decreases as the DHM content (*c*) increases (Figure 5, Group A). This is because DHM, as a small molecule, acts as a stabilizer for the emulsion by reducing the interfacial tension at the water–oil interface. It rapidly diffuses into the system, leading to the formation of finer droplets. Additionally, DHM exhibits potential surface activity, and increasing its dosage enhances the surface activity of the DHM/SPI complex, allowing for better adsorption at the oil–water interface. This reduces droplet aggregation and further decreases droplet size [25].

The increased content of DHM helps disperse the oil droplets. The rigid structure of DHM supports the gradual aggregation of the complex particles, forming a rigid three-dimensional network structure that disperses the oil droplets. This increases the viscosity of the emulsion and promotes the formation of a gel-like structure. The finding is consistent with Geng et al. [26], who observed that the droplet size of Pickering emulsions stabilized by DHM and HCS composite particles decreased with increasing DHM content. They also found that DHM additions limited the droplet size to a threshold range, stabilizing the emulsions. When the oil phase increases, the fixed DHM/SPI particle concentration in the high oil-phase system may no longer adequately cover the emulsification interface (Figure 5, Group B). This leads to difficulty in dispersing the oil phase into fine droplets, causing the emulsion to aggregate and increasing droplet size [55]. However, increasing the concentration of particlesin the emulsion systemallows for the formation of smaller droplets, as the enhanced coverage of the water–oil interface by DHM/SPI complexes leads to the formation of dense particle shells (Figure 5, Group C). This tighter packing of the droplets impedes aggregation, improving the emulsification effect [56]. Changes in ionic strength alter the adsorption behavior and interfacial properties of protein particles, causing more particles to adsorb to the oil–water interface and participate in stabilizing the emulsion. This results in a decrease in droplet size, which then stabilizes(Figure 5, Group D).

### 3.10. Zeta Potential Analysis of Emulsion

**Figure 6:**
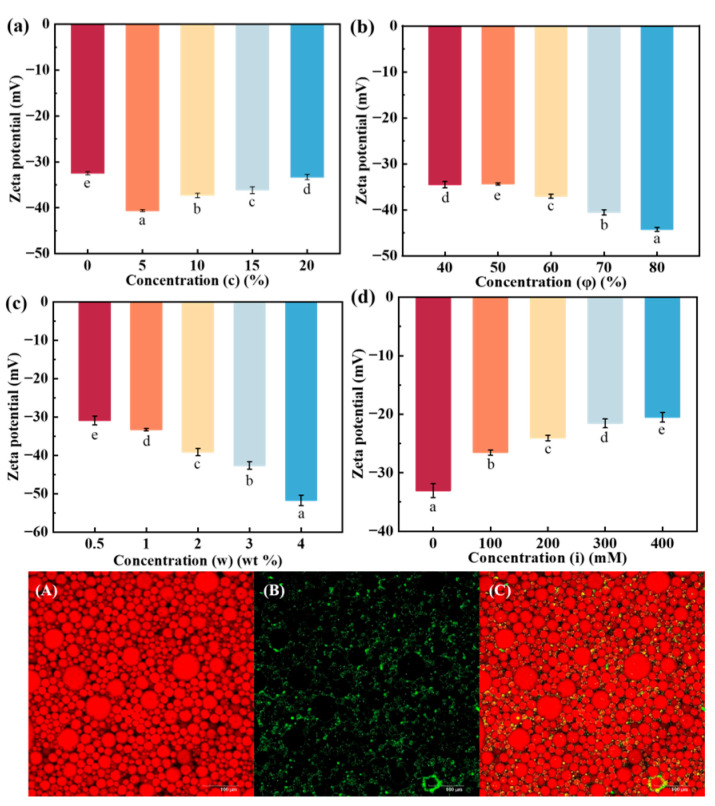
(**a**–**d**) Zeta potential data of Pickering emulsion gels prepared under different conditions; (**A**–**C**) CLSM images of stabilized emulsion gels of composite particles at *c* = 5%, *w* = 2%, *i* = 0, and *φ* = 70% ((**A**): Nile Red stained grease in red; (**B**): Nile Blue A stained DHM/SPI particles in green; (**C**): superimposed image of oil droplets with DHM/SPI complexes); scale bar: 100 μm. Different letters indicate significant differences at *p* < 0.05.

Zeta potential (ζ) provides insight into the charge characteristics of Pickering particles within the stabilized emulsion system, reflecting the charge-carrying condition of the emulsions. Higher absolute ζ values indicate stronger repulsion between droplets [57]. In the case of the Pickering emulsions studied, all ζ values were negative, echoing the negatively charged surface of the DHM/SPI composite particles of the stabilized emulsions mentioned in the previous section, which is the result of a combination of factors. For the emulsions, the adsorption of the DHM/SPI complex at the water–oil interface results in negatively charged oil droplet surfaces. Additionally, the partial dissociation of polar groups on the complex’s surface, along with the presence of electrolytes, can induce proper flocculation, electrostatic shielding at low concentrations, and bilayer compression at high concentrations, all of which further enhance the negative surface charge. As the DHM content (*c*) increased, the ζ value of the Pickering emulsion decreased from −32.5 mV to −40.6 mV at a DHM content of 5% but then rose again to −33.3 mV when the DHM content increased to 20%. This change in ζ potential can be attributed to the interaction between DHM and SPI, which caused a decrease in the ζ value. The increased DHM concentration led to SPI aggregation, covering the surface of the proteins with charged groups (Figure 6a). This coverage reduced the electrostatic repulsion between droplets, which in turn increased the ζ potential value [58]. Similar results were reported in emulsification systems regulated by young apple polyphenols (YAPs) interacting with SPI [59].

When the oil phase ratio was increased, the ζ potential values decreased. For oil phase volumes of 70% and 80%, the ζ potential values dropped to −40.5 mV and −44.23 mV, respectively (Figure 6b). These values corresponded to optimal emulsion stability and increased droplet packing for oil phase ratios of 70% and 80%. The increase in oil phase volume enhanced the interfacial adsorption of DHM/SPI particles, increasing the surface charge of the aggregated oil droplets and thereby raising the absolute ζ value. The increased addition of DHM/SPI particles leads to more particles being adsorbed at the oil–water interface, forming a denser interfacial layer. This enhances charge accumulation at the interface and increases repulsion between the particles, resulting in a higher absolute value of the potential. When the particle concentration (*w*) exceeds 2 wt%, the absolute value of ζ for the emulsion exceeds 40 mV, indicating excellent emulsion stability and a gel-like state (Figure 6c). Additionally, as ionic strength increased (Figure 6d), the absolute ζ value decreased. The electrostatic shielding effect caused by NaCl reduced the electrostatic repulsion between droplets, leading to a decrease in surface charge. As a result, the emulsions exhibited larger particle sizes and lower ζ values [60].

### 3.11. CLSM Observation of Emulsion

The microstructure of the Pickering emulsion stabilized by the DHM/SPI complex was further examined using confocal laser scanning microscopy (CLSM) (Figure 6A–C). The oil phase was stained with Nile red, and the resulting image revealed that the red oil droplets were distributed inside the droplets (Figure 6A). These rounded red oil droplets were independently dispersed in the black aqueous phase, typical of an O/W (oil-in-water) emulsion, consistent with the results of the contact angle measurements of the DHM/SPI complexes [61]. The DHM/SPI complexes were stained with Nile blue A, which highlighted them with green fluorescence. The tiny particles were uniformly distributed throughout the system (Figure 6B) and adsorbed onto the droplet surface, forming a spatial barrier that prevented the oil droplets from aggregating. Additionally, the DHM/SPI composite particles were dispersed in the aqueous phase, which helped prevent oil droplet agglomeration and contributed to the emulsion’s stability [62].

The CLSM test results demonstrated that DHM/SPI was effective in emulsifying the O/W-type Pickering emulsion. Based on these findings, we hypothesize the following trajectory of particles when DHM/SPI stabilizes Pickering emulsions: Initially, the composite particles are evenly dispersed in water to create a suspension. The oil phase is then added, and, after high-speed shear, the oil is uniformly dispersed into the aqueous phase, breaking into small droplets that mix with the DHM/SPI suspension. The particles then adsorb to the surface of the oil droplets through interactions between the droplets and shear force, creating a physical barrier that prevents aggregation and forms a mesh structure. Additionally, these particles bridge between droplets, forming a stable network that enhances the overall stability of the Pickering emulsion [63].

### 3.12. Rheological Behavior Analysis of Emulsion

**Figure 7:**
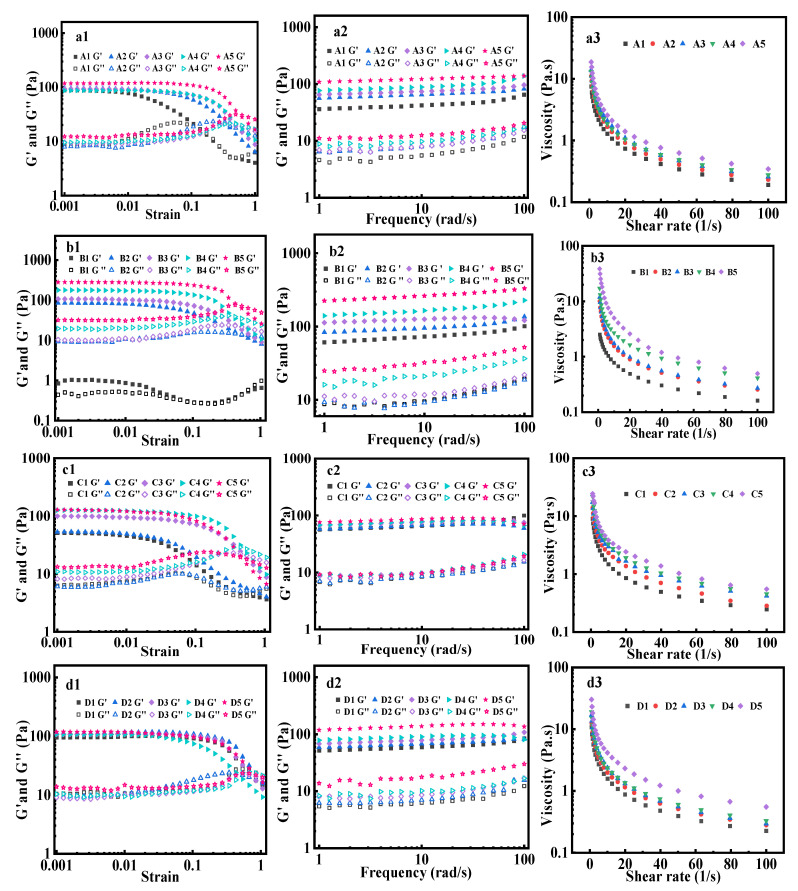
Rheological behavior of DHM/SPI Pickering emulsion prepared under different conditions: strain scan (**a1**–**d1**), frequency scan (**a2**–**d2**), and apparent viscosity (**a3**–**d3**).

Rheological tests were conducted on the emulsions to assess the material properties of the emulsion (Figure 7a1–d3). Strain scanning revealed that the storage modulus (G′) and loss modulus (G″) remained relatively constant within a small strain range (0.1%–1%), allowing the identification of the linear viscoelastic region (LVR) of the samples (Figure 7a1–d1). However, as the strain was increased beyond this range, both G′ and G″ decreased, indicating that the emulsion gel-like structure had been compromised. When the strain value was fixed at 1% within the LVR and frequency scanning was performed, the results showed that G′ and G″ differed by one order of magnitude for all emulsions, with G′ being significantly larger than G″ (Figure 7a2–d2). This indicates that the DHM/SPI Pickering emulsions exhibit excellent elastic-dominant behavior, characteristic of an emulsion gel-like structure. This behavior suggests that the emulsions have a certain resistance to deformation, allowing them to maintain stability over time and prevent droplet aggregation, thus protecting system inclusions from degradation [64]. Notably, when the frequency was increased from 1 rad/s to 100 rad/s, the G′ values for all emulsions remained stable, while G″ showed a gradual increase. The minimal dependency of both moduli on the frequency further reflected the strong stability of the emulsions in high-frequency oscillation modes [44].

In the study of DHM/SPI-stabilized Pickering emulsion systems prepared under different conditions, it is evident that increasing the amount of DHM added to the composite particles(*c*), the oil-to-water ratio(*φ*), the amount of DHM/SPI (*w*), and the ionic strength (*i*) results in increased moduli (G′ and G′′) and viscosity of the emulsions. The apparent viscosities of the emulsions decreased with increasing shear rate, demonstrating shear-thinning behavior due to the disruption of the emulsion structure under shear stress (Figure 7a3–d3). This behavior is characteristic of pseudoplastic fluids in non-Newtonian flow. As the shear rate increases, the non-covalent bonds maintaining the system, such as hydrogen bonding, hydrophobic interactions, and van der Waals forces, become more susceptible to breaking, leading to the disruption of the stable gel-like network structure. This results in the rearrangement or detachment of DHM/SPI particles from the droplet surface, weakening the repulsive interactions between droplets and reducing the viscosity [65]. Notably, increasing ionic strength raised the viscosity of the emulsion (Figure 7d3), with the highest viscosity observed at an ionic strength of 400 mM. This increase is likely due to the enhanced electrostatic shielding effect, which promotes particle aggregation, increases the interaction forces between droplets, and induces the formation of a stronger three-dimensional network structure. This results in greater emulsion stiffness and an increase in viscosity [66].

### 3.13. Lipid Peroxidation Analysis of Emulsion

**Figure 8:**
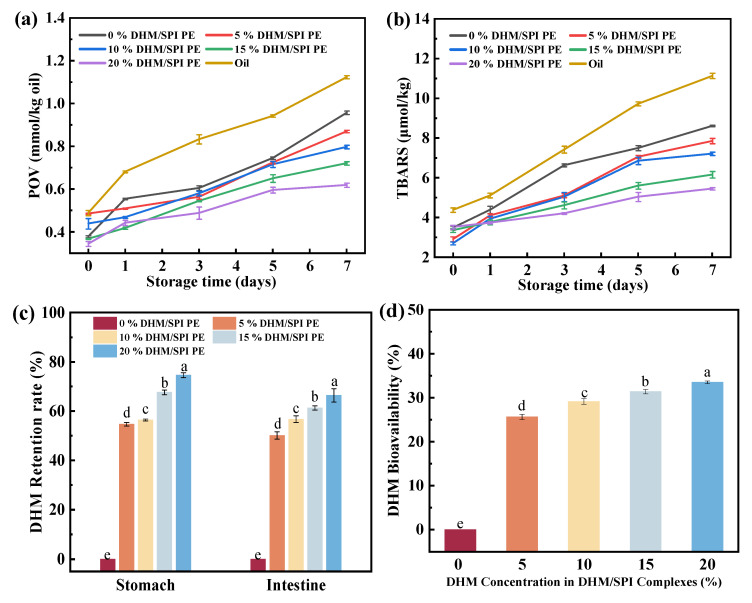
Peroxide values (**a**) and TBARS values (**b**) of Pickering emulsion gels stabilized by DHM/SPI complexes with different DHM additions (c = 0–20%) stored at 40 °C for 7 d; gastric and intestinal retention (**c**) and bioaccessibility (**d**) of DHM after *in vitro* digestive simulation of Pickering emulsions stabilized by DHM/SPI complexes with different DHM additions (*c* = 0–20%). The different letters indicate significant differences at *p* < 0.05.

To assess the inhibition of lipid peroxidation by DHM/SPI complexes, Pickering emulsions were prepared with varying DHM concentrations. High temperatures were used to accelerate the oxidative deterioration of lipids during storage. During the pre-storage period, the elevated temperature rapidly induced lipid oxidation, promoting the formation of oxidation products, which led to a sharp increase in peroxide value (POV) and thiobarbituric acid reactive substances (TBARSs). As storage time progressed, the oxidation rate gradually decreased (Figure 8a,b).

For oil-in-water emulsions, the rate and extent of lipid oxidation were influenced by the composition and structure of the oil–water interface. The phenolic hydroxyl groups of DHM can donate hydrogen atoms to neutralize free radicals generated during lipid oxidation, thus scavenging the radicals and inhibiting the oxidation chain reaction. Huang et al. [67] found that stabilizing Pickering emulsions with noncovalent compounds like quercetin, curcumin, and resveratrol in combination with rice protein slowed down lipid oxidation compared to the faster lipid oxidation observed in rice protein Pickering emulsions alone. Among these compounds, the quercetin/rice protein complex emulsion was the most effective in inhibiting lipid oxidation. This could be attributed to the numerous phenolic hydroxyl groups in quercetin and its low oil–water partition coefficient (Log *p* value).

### 3.14. In Vitro Simulated Digestion Analysis of Emulsion

The low bioavailability of the flavonoid compound DHM, typically ranging from 2 to 20%, may be attributed to its susceptibility to degradation in the gastrointestinal tract. This instability reduces the gastrointestinal retention of DHM, limiting its pharmacological effectiveness [68]. To evaluate the gastrointestinal retention and bioaccessibility of DHM after digestion with DHM/SPI Pickering emulsions, we prepared Pickering emulsions with varying DHM concentrations for *in vitro* simulated digestion experiments. The results demonstrated that the gastric and intestinal digestive retention of DHM in the Pickering emulsion increased with higher DHM content. This suggests that DHM is better retained in the gastric and intestinal environments after emulsion digestion, thereby enhancing its absorption in the small intestine (Figure 8c). This suggests that the gastric environment can better retain digested DHM, enhancing its small intestine absorption. The DHM/SPI complexes form a dense interfacial film on oil droplets, stabilizing the O/W Pickering emulsion. Under acidic conditions, the proteins clump together and act as a barrier while retarding pH changes in the system, protecting the DHM from digestion, maintaining the integrity and antioxidant activity of the polyphenols, and minimizing the loss of polyphenols as they reach the site of absorption. After digestion, the emulsion droplets lose integrity, reducing the number of complexes and leading to larger droplets. Phenolic compounds remain stable and do not decrease significantly in an acidic environment, and the interaction between proteins and polyphenols protects polyphenols from pH and digestive enzymes during gastrointestinal digestion, thus enhancing targeted delivery and absorption of polyphenols. Additionally, the preliminary XRD results confirmed that the crystalline structure of the DHM/SPI complex was altered, which, in turn, influenced the release and dissolution behavior of DHM. This phenomenon was also reported by Molaveisi et al. [69]. The use of DHM in the preparation of nanocochlea transforms DHM into a partially amorphous state, extending the release time and inducing structural changes. The controlled release occurs through a two-step diffusion process, resulting in an elevated release rate. The nanocochlea can effectively regulate both the release rate and quantity of DHM, maximizing its potential as a delivery system in the food industry. After digestion and absorption in the small intestine, the bioaccessibility of DHM, when added at 20% in Pickering emulsion, reached 33.51 ± 0.31%, indicating enhanced absorption efficiency (Figure 8d). Similarly, during the *in vitro* digestion of Pickering emulsions stabilized by soybean isolate protein/rutin complexes, the bioaccessibility of rutin remained stable at around 30% after digestion of emulsions prepared at different temperatures and homogenization rates. The trend of bioaccessibility changes remained consistent as digestion time was prolonged [28]. To confirm that the DHM in the emulsion was absorbed and utilized for physiological activity, Lyu et al. conducted *in vivo* experiments to investigate the effects of a DHM self-microemulsion system on mice fed a high-fat diet. This study found that the emulsion system improved the absorption rate of DHM, effectively suppressing increases in body weight and fat mass, as well as preventing non-alcoholic fatty liver disease in mice. Furthermore, the DHM self-microemulsion regulated and protected the mice’s physiological health by improving their intestinal flora [70]. Overall, Pickering emulsion enhances DHM absorption and protects it from degradation, thereby maximizing its physiological functions.

## 4. Conclusions

Food-grade Pickering emulsions hold significant potential for applications in food, pharmaceuticals, and materials development due to their stability, loading capacity, and safety. In this study, all DHM/SPI complexes exhibited hydrophilicity, with the largest contact angle of 66.82° observed for the complex containing 5% DHM. The Pickering emulsions stabilized by these complexes were of the O/W type and primarily exhibited an elastic gel-like network structure, behaving as a non-Newtonian fluid with a shear-thinning effect. DHM slows lipid oxidation, extending the emulsion’s shelf life. After *in vitro* simulated digestion, Pickering emulsions were able to exert a protective effect to protect DHM from degradation. The highest DHM bioaccessibility in the emulsion stabilized by 20% DHM/SPI complex was 33.51 ± 0.31%. This improvement in DHM bioaccessibility addresses its initial low bioaccessibility, opening greater potential for applications. The combination of DHM and SPI overcomes the limitations of single biobased particulate emulsifiers, offering broader applications in disease prevention and functional substance delivery. The present study introduces a novel strategy to enhance the effective use of DHM, which offers a promising application for more functional ingredients with poor solubility, like DHM. DHM is a natural ingredient renowned for its outstanding biological activity. Despite its remarkable physiological functions, such as antioxidant, anti-inflammatory, and anti-tumor properties, DHM exhibits poor solubility in both water and oil, as well as limited dispersion, which restricts its potential applications. By preparing a complex of DHM with SPI, we achieved a stable dispersion of composite particles in the system, which opens new avenues for studying functional ingredients with limited dispersibility. DHM is increasingly valued as a medicinal ingredient in the food and pharmaceutical industries, which are derived from *Ampelopsis grossedentata*. Moving forward, we will explore further applications of DHM in emerging systems by incorporating oil-soluble substances in the oil phase, enabling a synergistic effect with DHM. We believe that these studies will lead to more innovative and promising applications in the future.

## Figures and Tables

**Table 1:** Grouping table for preparing Pickering emulsions.

Sample Group	DHM Content in Complexes (*c*/%)	Concentration of Complex (*w*/%)	Oil Phase Fraction (*φ*/%)	Ionic Strength (*i*/mM)
Group A (A1–A5)	0–20%	2%	70%	0 mM
Group B (B1–B5)	5%	2%	40–80%	0 mM
Group C (C1-C5)	5%	0.5–4%	70%	0 mM
Group D (D1–D5)	5%	2%	70%	0–400 mM

**Table 2:** Color parameters of complexes with different DHM additions.

Type	L*	a*	b*	∆E	WI
0% DHM/SPI	79.63 ± 1.01 ^a^	0.07 ± 0.06 ^e^	10.75 ± 0.18 ^d^	80.35 ± 0.98 ^a^	76.97 ± 0.98 ^a^
5% DHM/SPI	60.26 ± 0.36 ^b^	1.84 ± 0.15 ^d^	17.99 ± 0.81 ^a^	62.92 ± 0.11 ^b^	56.33 ± 0.67 ^b^
10% DHM/SPI	57.58 ± 0.62 ^c^	3.70 ± 0.26 ^c^	12.68 ± 0.31 ^c^	59.07 ± 0.65 ^c^	55.56 ± 0.53 ^c^
15% DHM/SPI	56.43 ± 0.66 ^cd^	5.38 ± 0.34 ^a^	10.33 ± 0.44 ^d^	57.62 ± 0.56 ^d^	54.90 ± 0.75 ^d^
20% DHM/SPI	55.32 ± 0.45 ^d^	4.52 ± 0.23 ^b^	14.87 ± 0.42 ^b^	57.47 ± 0.42 ^d^	52.70 ± 0.48 ^e^

In this table, L*, a*, and b* represent the luminance, red to green, and yellow to blue of the prepared DHM/SPI complexes, respectively. In addition, ∆E and WI represent the total color difference and whiteness index of the DHM/SPI complexes, respectively. Different lowercase letters in the same column indicate significant differences (*p* < 0.05).

**Table 3:** Creaming index (CI) of Pickering emulsions prepared under different conditions during the storage period.

Group	Name	CI %				
		Day 0	Day 1	Day 5	Day 10	Day 15
A	A1	0	0	0	0	0
	A2	0	0	0	0	0
	A3	0	0	0	0	0
	A4	0	0	0	0	0
	A5	0	0	0	0	0
B	B1	0	39.43 ± 0.42	42.10 ± 0.35	48.18 ± 0.36	48.55 ± 0.46
	B2	0	22.44 ± 0.82	28.75 ± 0.46	34.04 ± 0.39	35.26 ± 0.11
	B3	0	10.98 ± 0.63	14.26 ± 0.48	19.22 ± 0.59	22.42 ± 0.46
	B4	0	0	0	0	0
	B5	0	0	0	0	0
C	C1	0	13.02 ± 0.57	17.79 ± 0.45	19.02 ± 0.73	20.28 ± 0.60
	C2	0	6.28 ±0.29	10.79 ± 0.33	14.55 ± 0.72	16.22 ± 0.71
	C3	0	0	0	0	0
	C4	0	0	0	0	0
	C5	0	0	0	0	0
D	D1	0	0	0	0	0
	D2	0	0	0	0	0
	D3	0	0	0	0	0
	D4	0	0	0	0	0
	D5	0	0	0	0	0

## Data Availability

The original contributions presented in this study are included in the article; further inquiries can be directed to the corresponding author.
